# Using digital health to facilitate compliance with standardized pediatric cancer treatment guidelines in Tanzania: protocol for an early-stage effectiveness-implementation hybrid study

**DOI:** 10.1186/s12885-020-6611-3

**Published:** 2020-03-29

**Authors:** Lavanya Vasudevan, Kristin Schroeder, Yadurshini Raveendran, Kunal Goel, Christina Makarushka, Nestory Masalu, Leah L. Zullig

**Affiliations:** 1 Department of Family Medicine and Community Health, Duke School of Medicine, 2200 W. Main Street, Durham, NC 27710 USA; 2 Duke Global Health Institute, 310 Trent Drive, Durham, NC 27710 USA; 3 Department of Pediatrics, Division of Hematology/Oncology, Duke University Medical Center, 2301 Erwin Rd, Durham, NC 27710 USA; 4 Bugando Medical Centre, Wurtzburg Road, Mwanza, Tanzania; 5 Duke School of Medicine, 8 Searle Center Drive, Durham, NC 27710 USA; 6 Department of Population Health Sciences, 215 Morris St, Durham, NC 27701 USA; 7 Center of Innovation to Accelerate Discovery and Practice Transformation (ADAPT), Durham Veterans Affairs Health Care System, 508 Fulton St, Durham, NC 27705 USA

**Keywords:** Digital health, Pediatric cancer, Protocol-driven treatment, Treatment abandonment, Retinoblastoma, Burkitt lymphoma, Low- and middle-income countries, Tanzania, Healthcare provider decision support, Client health records

## Abstract

**Background:**

In high-income countries (HICs), increased rates of survival among pediatric cancer patients are achieved through the use of protocol-driven treatment. Compared to HICs, differences in infrastructure, supportive care, and human resources, make compliance with protocol-driven treatment challenging in low- and middle-income countries (LMICs). For successful implementation of protocol-driven treatment, treatment protocols must be resource-adapted for the LMIC context, and additional supportive tools must be developed to promote protocol compliance. In Tanzania, an LMIC where resource-adapted treatment protocols are available, digital health applications could promote protocol compliance through incorporation of systematic decision support algorithms, reminders and alerts related to patient visits, and up-to-date data for care coordination. However, evidence on the use of digital health applications in improving compliance with protocol-driven treatment for pediatric cancer is limited. This study protocol describes the development and evaluation of a digital health application, called mNavigator, to facilitate compliance with protocol-driven treatment for pediatric cancer in Tanzania.

**Methods:**

mNavigator is a digital case management system that incorporates nationally-approved and resource-adapted treatment protocols for two pediatric cancers in Tanzania, Burkitt lymphoma and retinoblastoma. mNavigator is developed from an open-source digital health platform, called CommCare, and guided by the Consolidated Framework for Implementation Research. From July 2019–July 2020 at Bugando Medical Centre in Mwanza, Tanzania, all new pediatric cancer patients will be registered and managed using mNavigator as the new standard of care for patient intake and outcome assessment. Pediatric cancer patients with a clinical diagnosis of Burkitt lymphoma or retinoblastoma will be approached for participation in the study evaluating mNavigator. mNavigator users will document pre-treatment and treatment details for study participants using digital forms and checklists that facilitate compliance with protocol-driven treatment. Compliance with treatment protocols using mNavigator will be compared to historical compliance rates as the primary outcome. Throughout the implementation period, we will document factors that facilitate or inhibit mNavigator implementation.

**Discussion:**

Study findings will inform implementation and scale up of mNavigator in tertiary pediatric cancer facilities in Tanzania, with the goal of facilitating protocol-driven treatment.

**Trial registration:**

The study protocol was registered in ClinicalTrials.gov (NCT03677128) on September 19, 2018.

Contributions to the literature

- Implementation of potentially sustainable, technology-based interventions is limited in low-and middle-income countries. mNavigator demonstrates how a digital case management system can be used to support implementation of resource-adapted treatment protocols in global oncology, with an eye toward sustainability.
- mNavigator relies on a strong theoretical framework, the Consolidated Framework for Implementation Research, to inform user-centered design, implementation, and evaluation.
- mNavigator is designed to be agnostic of health care system or country. While it is designed for use with clinical practice guidelines adapted for Tanzania, it could be adapted again and/or disseminated to other countries and contexts.

## Background

In high-income countries (HICs), protocol-driven treatment has led to substantial improvements in survival among pediatric cancer patients by reducing uncertainty in clinical decision-making, creating uniformity in the approach to diagnosis and treatment, and ensuring consistency across providers [1–4]. However, over 85% of the 400,000 children newly diagnosed with cancer each year live in low- and middle-income countries (LMICs) where differences in infrastructure, supportive care, and human resources limit implementation of protocol-driven treatment [4]. These challenges in LMICs necessitate protocol adaptation for available resources to achieve successful implementation of protocol-driven treatment. Yet, in many LMIC settings where resource-adapted protocols are available, suboptimal protocol compliance contributes to treatment abandonment, further exacerbating the 60% survival disparity gap between HICs and LMICs. The use of supportive tools can facilitate compliance with protocol-driven treatment by standardizing clinical decision-making, and incorporation of decision support, checklists, and improved data use. However, in LMICs, instances of, and evidence on the effectiveness of such supportive tools is lacking.

Digital health applications have been used as tools to support providers with implementation of standardized protocols for the integrated management of childhood illnesses in Tanzania, HIV care in South Africa, and antenatal care in Nigeria [5–11]. In the case of integrated management of childhood illnesses, provider compliance with the digital protocol increased by up to 30% compared to the use of a paper-based protocol [5]. In addition to the impact on protocol-driven treatment, digital health applications have been applied in low-resource settings to facilitate task shifting, improve work planning and coordination between providers, as well as enhance the performance of health workers [12–15]. These data support the use of digital health applications to improve compliance with protocol-driven treatment [16–18].

The goal of this early-stage effectiveness-implementation hybrid study is to develop a digital case management system, called mNavigator, to facilitate protocol-driven treatment for pediatric cancer, and evaluate its preliminary effectiveness in a tertiary care setting in Tanzania. Currently, resource-adapted treatment protocols for two pediatric cancers, Burkitt lymphoma and retinoblastoma, are approved for use at all pediatric cancer centers by the Tanzanian Ministry of Health, Community Development, Gender, Elderly and Children. However, compliance with these treatment protocols is low in pediatric cancer centers in Tanzania, making this an ideal LMIC setting for testing a digital health system for supporting protocol compliance. To our knowledge, mNavigator is the first digital case management system leveraging mobile devices and being developed for improving protocol compliance in pediatric cancer in LMICs.

## Methods/design

The elements of the mNavigator system are reported below consistent with the Template for Intervention Description and Replication (TIDieR) checklist (see Table 1), the SPIRIT checklist for protocols (Additional file 1 Table S1), and the World Health Organization trial registration dataset (Additional file 1 Table S2).

**Table 1:** The TIDieR (Template for Intervention Description and Replication) Checklist*: Information to include when describing an intervention and the location of the information

Item number	Item	Where located **	
		Primary paper (page or appendix number)	Other ^†^ (details)
	BRIEF NAME		
1.	Provide the name or a phrase that describes the intervention.	____8________	______________
	WHY		
2.	Describe any rationale, theory, or goal of the elements essential to the intervention.	___13, Additional file 1 Table S4_______	_____________
	WHAT		
3.	Materials: Describe any physical or informational materials used in the intervention, including those provided to participants or used in intervention delivery or in training of intervention providers. Provide information on where the materials can be accessed (e.g. online appendix, URL).	___9-11_____	_____________
4.	Procedures: Describe each of the procedures, activities, and/or processes used in the intervention, including any enabling or support activities.	_13–18, table 2_	_____________
	WHO PROVIDED		
5.	For each category of intervention provider (e.g. psychologist, nursing assistant), describe their expertise, background and any specific training given.	__12_____	_____________
	HOW		
6.	Describe the modes of delivery (e.g. face-to-face or by some other mechanism, such as internet or telephone) of the intervention and whether it was provided individually or in a group.	__12-13________	_____________
	WHERE		
7.	Describe the type(s) of location(s) where the intervention occurred, including any necessary infrastructure or relevant features.	___9, Additional file 1 Table S3______	_____________
	WHEN and HOW MUCH		
8.	Describe the number of times the intervention was delivered and over what period of time including the number of sessions, their schedule, and their duration, intensity or dose.	____12_______	_____________
	TAILORING		
9.	If the intervention was planned to be personalised, titrated or adapted, then describe what, why, when, and how.	_14, figure 1___	_____________
	MODIFICATIONS		
10.^a^	If the intervention was modified during the course of the study, describe the changes (what, why, when, and how).	_____N/A_____	_____________
	HOW WELL		
11.	Planned: If intervention adherence or fidelity was assessed, describe how and by whom, and if any strategies were used to maintain or improve fidelity, describe them.	_assessed by data from app (protocol compliance), Tables 3&4_	_____________
12.^a^	Actual: If intervention adherence or fidelity was assessed, describe the extent to which the intervention was delivered as planned.	____N/A______	_____________

^a^ These items are not relevant to the protocol and cannot be described until the study is complete

### Study aims

This early-stage effectiveness-implementation hybrid study has two primary aims:

Aim 1. To develop mNavigator by adapting an open-source digital health case management platform, CommCare, to incorporate protocol-driven treatment for pediatric cancer.

Aim 2. To evaluate the effectiveness of mNavigator for improving provider compliance with protocol-driven treatment for pediatric cancer and reducing treatment abandonment.

A secondary aim of the study is to understand factors that facilitate or inhibit the implementation of mNavigator in tertiary care settings for pediatric cancer.

### Study setting

The study will be implemented at Bugando Medical Centre (BMC) in Mwanza, Tanzania. BMC serves a catchment area of 15 million people and is one of three tertiary cancer centers in Tanzania that treat pediatric cancer patients. In 2019, the oncology unit at BMC comprises 2 medical oncologists, 1 radiation oncologist, 1 junior medical officer, 10 nurses, 2 pediatric patient navigators, and 1 clinic coordinator. Annually, approximately 150 new pediatric patients are diagnosed with cancer at BMC. The Tanzanian Ministry of Health Community Development, Gender, Elderly and Children collaborated with representatives from each of the three tertiary pediatric cancer centers in Tanzania to develop a protocol–treatment consensus for two of the most common national pediatric cancer diagnoses -- Burkitt lymphoma (BL) and retinoblastoma (Rb). These diagnoses constitute 35% of children with cancer presenting to BMC. Despite the introduction of these guidelines, provider compliance with these guidelines is less than 20%. (Kristin Schroeder, Personal communication).

### Intervention

#### Materials

Materials specific to intervention development are described below.

- Treatment protocols: International pediatric cancer consortiums have developed resource-adapted treatment protocols specifically for use in LMICs [19–22]. The study uses resource-adapted treatment protocols for Burkitt lymphoma and retinoblastoma, which have already been approved for use at three pediatric cancer centers by the Tanzanian Ministry of Health Community Development, Gender, Elderly and Children [23].
- Software and subscription plan: mNavigator will be developed using CommCare, a highly validated, HIPAA-compliant, open-source digital health platform developed by Dimagi Inc. [24, 25] This extensible and modular platform includes an existing module for tracking individuals through a continuum of service delivery that can be customized for the proposed application to manage pediatric cancer care [26]. The CommCare platform has two core components: a mobile application and CommCareHQ. CommCare mobile application runs on a mobile phone or tablet, and is built on a decision and logic-processing platform that can support oncology providers and staff by providing critical data-quality checks based on patient data and calculations at each point of service throughout treatment. CommCareHQ is a cloud-based system, which allows application development, data management and reporting. The application builder enables complex branching logic and data validation suitable for the implementation of a standardized protocol. The application works offline, making its use highly feasible in settings with low connectivity. Access to the CommCare platform is via a subscription plan with tiered pricing. For this study, the Pro plan was purchased ($500/month).
- Training: All study staff participating in the development of mNavigator completed two online training modules on the Dimagi Academy website (CommCare fundamentals and CommCare application building) prior to accessing CommCare HQ.
- CommCare accounts: Each study staff member created an account to log in to CommCare HQ. Account creation and access controls are managed centrally by an admin user.
- Hardware: mNavigator will be deployed on Android tablets. For this study, mNavigator was deployed on Samsung Galaxy Tab. A devices. Since the system is hosted on Dimagi’s servers and included in the subscription service, additional hardware related to data storage was not needed for this study or for future routine clinical use.

A list of resources is described in Additional file 1 Table S3. Resources are described as existing (available irrespective of study status) or study-supported (potentially not sustainable post-study).

#### Intervention components

mNavigator comprises four key modules:

1. Pre-diagnosis module: This includes data entry forms that enable registration of new patients, collection of socio-demographic data and clinical history, entry of laboratory and imaging results at presentation, and assignment of a working diagnosis.
2. Burkitt lymphoma (BL) module: This includes data entry forms specific to patients diagnosed with Burkitt lymphoma to document cancer staging, planned treatment (including details of the dose and timing of for each chemotherapy cycle), end of therapy evaluation, and follow up visit planning. Throughout the forms in this module, Burkitt lymphoma treatment guidelines are incorporated as prompts for data entry, computation of relevant lab values, adjustment of chemotherapy regimen, scheduling of chemotherapy cycles, and post-treatment follow up.
3. Retinoblastoma (Rb) module: This module is similar to the Burkitt module, except that it is specific to patients diagnosed with retinoblastoma and incorporates the specific treatment guidelines for retinoblastoma.
4. Non-BL/Rb module: This module includes data entry forms for patients who are not diagnosed with Burkitt lymphoma or retinoblastoma. The forms do not track the treatment of the patients or incorporate treatment guidelines. Rather, they enable tracking of patient demographics and outcomes.

#### Intervention users

Four users (1 junior physician, 2 patient navigators, and 1 clinic coordinator) will be trained to use mNavigator at BMC. In addition to the physician, one patient navigator has medical training as a clinical officer. The remaining two users have training in social work. All four have worked in the oncology department for at least 2 years and were chosen as the intended users of mNavigators since they are currently responsible for coordinating clinical care for pediatric cancer patients at BMC, and hence, frequently interact with patients and their families at the hospital. They are comfortable with smartphone technology (they own and use personal smartphones) and, as part of current job responsibilities, are knowledgeable with accessing online databases in cloud based systems.

#### Mode of delivery

The users will access mNavigator on an Android tablet (Samsung Galaxy Tab. A). To access mNavigator, users will log into the CommCare application then select mNavigator from a menu of applications. CommCare supports offline log in and data collection.

#### Intervention delivery

The users will use mNavigator during one-on-one interactions with patients and their caregivers. At the first interaction with each cancer patient, the users will register the patient. During subsequent interactions, users will:

- Enter pre-diagnostic labs and imaging tests.
- Assign patients based on physician preliminary clinical evaluation to BL, Rb, or non-BL/Rb cohorts for further assessment.
- Complete and review pre-treatment staging and laboratory checklists for patients with preliminary Rb or BL diagnosis to facilitate protocol compliance.
- Deliver cancer educational information in video format.
- Review treatment guidelines with decision support algorithms to facilitate care coordination between mNavigator users and prescribing physicians.
- Enter information on changes in treatment plan, including referrals to outside hospitals, second line treatment, or palliation.
- Follow-up with patients to record health related outcomes (on treatment, off therapy, relapsed disease, etc.) and vital status.

### Theoretical framework for intervention development and evaluation

Our intervention development is guided by the Consolidated Framework for Implementation Research (CFIR) and our evaluation is informed by CFIR and RE-AIM [27, 28]. This study will address characteristics of the:

1. Outer setting (e.g., patients’ needs and resources, diagnosis delays, test availability);
2. Inner setting (e.g., compatibility of the protocols with the existing workflows at BMC, organizational readiness to change),
3. Individuals (e.g., providers’ self-efficacy for using the protocols, acceptability of intervention), and
4. Intervention (e.g., using evidence-based protocols; low complexity of intervention design) in this project.

Details of how the study will address the characteristics listed above are presented in Additional file 1 Table S4.

### Study activities

Study activities are summarized in Table 2. The implementation process using CFIR comprises four iterative steps: Plan, Engage, Execute, Reflect and evaluate. As described below, these four iterative steps are incorporated throughout mNavigator development and evaluation.

**Table 2:** Summary of study activities using the Consolidated Framework for Implementation Research process

CFIR phase	Activities	Tasks
PLAN	Workflow mapping	Existing clinical workflows Provider tasks Patient navigator tasks
	Form development	Translation of clinical workflows and national treatment guidelines to data entry forms Programming in CommCare HQ
ENGAGE	Quality assurance	Personas De-identified patient records Iterative testing and updates
	Usability testing	System usability score Think aloud method
EXECUTE	In-country training	Training on mNavigator In-country capacity building for sustainability
	Implementation in routine clinical use	Supported launch Full launch
REFLECT AND EVALUATE	Implementation- effectiveness hybrid design	Clinical effectiveness System evaluation Implementation factors

### Study phase 1: intervention development

The four study activities during mNavigator development are:

1. Workflow mapping and form development,
2. Form programming in CommCare
3. Quality assurance.
4. Usability testing

#### Activity 1

**Fig. 1:**
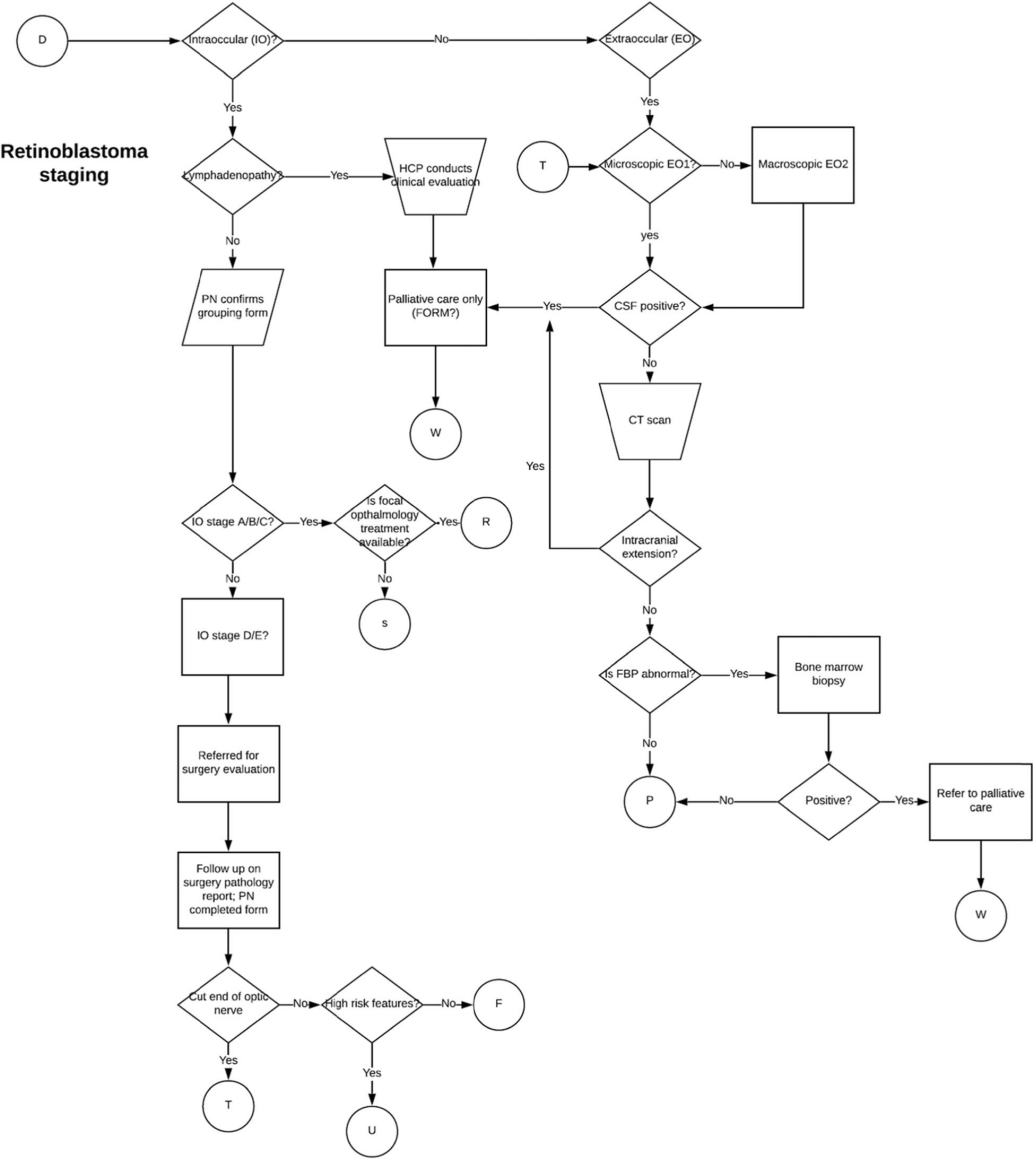
Detailed draft workflow for retinoblastoma staging incorporating clinical workflows at BMC and the nationally-approved resource-adapted standardized treatment protocol

*Workflow mapping:* During this stage, a pediatric cancer expert (KS) and a digital health expert (LV) led the development of workflow diagrams for mNavigator. The workflow diagrams were created using LucidChart Pro (www.lucidchart.com), and reflected the current clinical workflows at BMC as well as the nationally-approved, resource-adapted protocols for Burkitt lymphoma and retinoblastoma. Workflow diagrams were updated based on feedback from other study team members. Workflows attempted to capture all steps that mNavigator users would go through with pediatric cancer patients, beginning from patient registration and ending in an outcome form. As an illustration, the draft workflow for retinoblastoma staging is shown in Fig. 1. KS and LV developed a list of forms to document workflow steps and patient information. Over fifty forms were built out using Microsoft Word by KS and refined with input from study team members to mimic the eventual data entry prompts (including question type, skip logic, display logic, calculations, etc.) in mNavigator.

#### Activity 2

**Fig. 2:**
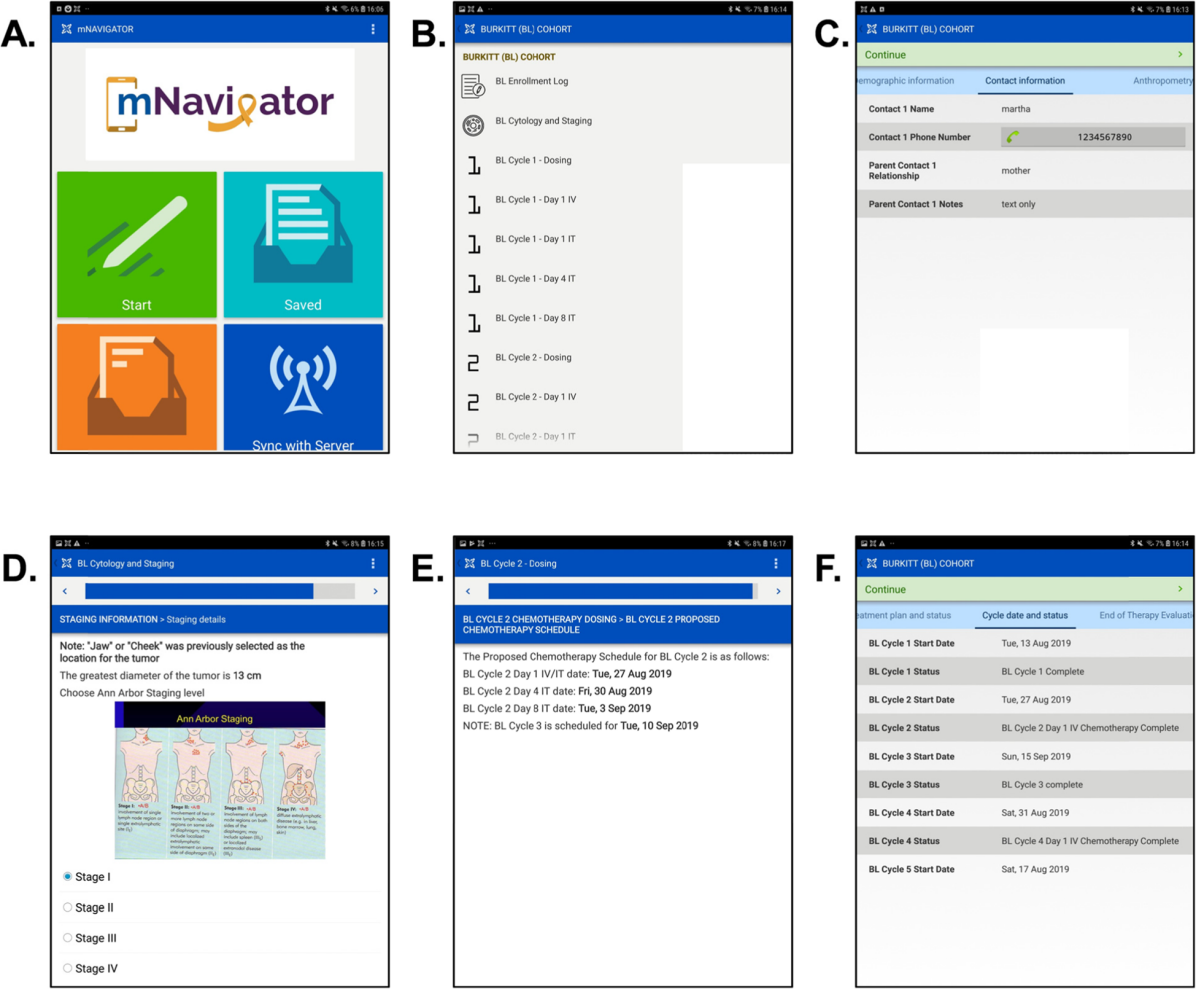
mNavigator user interface draft. **a**. mNavigator home screen. **b**. List of forms built in for Burkitt lymphoma patients. **c**. Example case detail showing contact information of a fictitious patient. **d**. Example of data entry question on tumor staging with pictorial support. **e**. Illustration of automated calculation of next chemotherapy cycle dates. **h**. Display example of chemotherapy cycle status

*Form programming:* A three-member programming team (LV, YR, KG) programmed the forms in the mNavigator application using CommCareHQ form builder. A dedicated project manager from Dimagi Inc. was assigned to the project as part of a 6-month advisory services contract. The project manager worked closely with the researchers to navigate any programming issues, assist with programming complex logic or calculations, and provide other consultation as necessary for mNavigator development. For each form, one programmer was assigned to be the primary builder, while a second programmer reviewed the build and made any necessary adjustments. Any modifications to the forms were discussed by the team before being implemented on CommCare. Figure 2 shows screenshots of the draft mNavigator user interface.

#### Activity 3

*Program Quality Assurance:* A quality assurance plan was implemented to check mNavigator for comprehensiveness of patient scenarios and clinical workflows, accuracy of clinical recommendations, and alignment with treatment guidelines. Steps in the quality assurance plan included:

1. Development of fictitious personas to simulate patients and most common workflow pathways, and test programmed decision logic. Details of personas included socio-demographic characteristics, clinical history, cancer diagnosis and staging, and treatment plan.
2. Testing the app for errors in flow or output using personas. Details of the personas were entered into mNavigator to assess the application flow, as well as to assess if calculations and recommendations being made are correct based on the standardized treatment protocol. An example of a correct application flow was for mNavigator to assign a patient to the Burkitt lymphoma module when a diagnosis of Burkitt lymphoma was entered in the diagnosis form. Any errors or areas for improvement were documented as detailed notes or checklists and used to inform revisions.
3. Testing the app for errors in flow or output using historical patient data. mNavigator was further evaluated using historical patient data to assess the application flow, as well as to assess if calculations and recommendations being made are correct based on the standardized treatment protocol. Any errors or areas for improvement were documented as notes.

#### Activity 4

*Usability testing:* Research staff introduced approximately 15 BMC personnel to mNavigator during a study launch event in July 2019. Attendees were BMC health professionals who provide routine clinical care for pediatric cancer patients including patient navigators, clinical coordinators, health providers and other clinical staff as well as non-clinical staff and other key stakeholders whose buy-in was necessary for the successful implementation of mNavigator. One BMC staff member with database management and information technology skills was trained on how to further customize, deploy and manage mNavigator. A post-usability survey with the four mNavigator users was used to assess system usability (using the System Usability Scale), relative advantage over standard of care, acceptability and satisfaction. Additional feedback on system features received during the study launch was also documented as notes.

### Phase 2: intervention evaluation

#### Study activities

Training and usability testing will be followed by supported implementation and evaluation (early-stage effectiveness-implementation trial).

#### Informed consent

mNavigator will be used as the standard of care for patient intake and outcome tracking of pediatric cancer patients at BMC. All pediatric cancer patients at BMC will be registered and tracked in mNavigator. For research purposes, research staff (e.g., research coordinator, mNavigator users, etc.) will consent caregivers of any patients who receive a clinical diagnosis of Rb or BL for tracking their treatment information. Data for only those providing informed consent will be used in the research study (with the exception of historical data). For consenting patients, mNavigator will be used for treatment management with a typical treatment duration of 3 months for patient with BL and 4 months for patient with Rb.

#### Data collection

There will be mixed methods data collection including semi-structured qualitative interviews and a quantitative survey including validated measures such as Organizational Readiness for Implementing Change (ORIC) [29].

##### Quantitative data collection

To measure compliance with standardized pediatric oncology protocols, we will use personal and clinical data points routinely collected as part of clinical visits along with the data entered into mNavigator.

We will compare treatment protocol compliance between BL/Rb retrospective patients (treated between 2015 and 19 when standardized treatment protocols for BL and Rb were introduced at BMC, but before introduction of mNavigator) and BL/Rb prospective patients (treated using mNavigator). To collect retrospective medical record data, trained research staff will abstract medical data into mNavigator from paper records for patients diagnosed with BL and Rb between 2015 and 19. Items abstracted will include as many data points available in paper records that are included in mNavigator.

To assess factors that may facilitate or inhibit implementation of the system and inform scale-up and design of future studies, we will periodically conduct observations, or use surveys and/or checklists to collect data related to the following areas:

- Technical functionality (such as content, time to complete forms)
- Technical stability (network connectivity, server downtime, failure and errors; issues with quality of data and system, device damage)
- Fidelity and quality of system implementation. These data will help us assess and describe the fidelity of the intervention (how mNavigator was used in practice and whether protocol steps were followed

##### Qualitative data collection

We will invite mNavigator users to complete a 30–45 min in-depth interviews to discuss system acceptance and usability, and satisfaction. Using the validated ORIC measure, we may also revisit the degree of change in readiness and commitment over time to use mNavigator and change in efficacy, a belief in the capacity at BMC to implement mNavigator.

We will also reach out to parents or caregivers of pediatric oncology patients to conduct in-depth qualitative interviews to explore factors that may contribute to treatment abandonment (barriers and facilitators to initiating or completing treatment). Participants will be able to choose to complete the interview in Swahili or English. Interviews will be transcribed verbatim. Those interviews conducted in Swahili will be translated into English.

We will document activities contributing to increased research capacity at BMC. Examples of research capacity include: (a) technology transfer and research capacity for implementation of digital health interventions among BMC investigators through collaborations with Duke and Dimagi Inc.; (b) continued development of research management capacity through weekly conference calls between project coordinators regarding budget management, quality assurance oversight, and local staff leadership. We will also document the process of training and ongoing support provided to mNavigator users.

### Data validation and audit

Data validation is built into mNavigator in the form of required responses, checks for response length and format, and decision support. The study PI (KS) and the junior medical officer will complete full audits of the first 5 enrolled BL and/or Rb patients whose treatment is tracked using mNavigator. Subsequently, they will audit records of 1 in every 10 patients. Any errors in mNavigator programming will be fixed on an ongoing basis.

### Participants

The following two groups of participants will be identified and screened for eligibility.

1. *BMC health professionals and staff:* We will approach BMC personnel, both who will directly use mNavigator and/or those whose work will be impacted by mNavigator, to offer enrollment in the study to help test the usability of the mNavigator system or provide general feedback prior to implementation and during implementation. BMC personnel will include health professionals who provide routine clinical care for pediatric cancer patients such as patient navigators, clinical coordinators, health providers and other clinical staff as well as non-clinical staff and other key stakeholders whose buy-in will be necessary for the successful implementation of mNavigator. Health professionals and staff will be identified to participate in this study based on the following inclusion criteria:

- Must be a health provider or staff working at BMC who provide care or support clinical care for cancer patients at BMC (medical oncologists, radiation oncologists, nurses, patient navigators, clinical coordinators, among others), or other key stakeholder.
- Must be 18 years or older at the time of informed consent.

2. *Parents or caregivers and their child who is a BMC pediatric oncology patient with diagnosis of BL or Rb*

As part of standard of care for patient intake, mNavigator will be used to register new BMC pediatric oncology patients yearly. Over the course of 1 year, patients with a diagnosis of BL or Rb will be followed for the duration of treatment (typically 3 months for BL and 4 months for Rb) using mNavigator. Eligible participants are:

- Patients with suspected or known diagnosis of either BL and Rb
- Patients younger than 18 years of age at enrollment

### Informed consent

Since all patients enrolled in the study will be children younger than 18 years old at the time of diagnosis, written consent will be obtained from parent, guardian or caregiver (see Additional file 2 for example consent). Assent will be sought for children who are 6 years old or older. Patients who turn 18 years of age during active study participation will be re-consented as adults. After explaining the purpose of the study, as well as the process, consent will be obtained in writing or verbally (with thumbprint in the presence of a literate witness), depending on participant’s literacy. Comprehension of the information provided will be ensured by asking potential participants if they completely understand the project aim and process. Research staff will also ask participants to repeat, in their own words, what they understand about the research study and how we are asking them to participate. These methods to ensure comprehension and avoid unintentional coercion will be taught to research staff prior to conducting any consents.

### Sample size and recruitment

Sample size estimates are based on the patient volume at BMC. Based on prior experience, approximately 150 new pediatric patients present each year for cancer management at BMC. Of these, approximately 50 patients are anticipated to have a diagnosis of BL or Rb. All new patients will be registered in mNavigator, and all patients with BL and Rb who provide informed consent will be tracked in the system for treatment management. Historical data is anticipated to be available for approximately 200 BL and Rb patients from 2015 to 2019 (i.e., 50 records/year). All available historical data will be used in the comparator arm. Similarly, approximately 15 health professionals at BMC are identified as individuals directly or indirectly impacted by mNavigator, all of whom will be approached for study participation. All prospective participants will be approached in person to provide study information and invite participation.

To ensure thematic saturation of qualitative data and that diverse perspectives are heard, we will complete an interview with parents of at least 12 BL and 12 Rb patients. Interviews may be recorded using an encrypted digital device.

### Outcome measures

Study outcomes are summarized in Tables 3 and 4. Measurement of study outcomes is guided by the RE-AIM framework. In mNavigator, we will collect data points that will allow us to measure outcomes in the following domains:

- Reach – e.g., proportion of eligible patients for whom protocol was used.
- Effectiveness – e.g., proportion of cases that abandoned care, with treatment completion and time from hospital presentation to confirmed diagnosis
- Adoption – e.g., proportion of providers who use the protocol, provider acceptability and satisfaction with mNavigator content, ease of delivery and credibility
- Implementation/Compliance – e.g., proportion of protocol steps completed per patient
- Maintenance (measured in future studies)

**Table 3:** Summary of outcomes related to intervention effectiveness and their measurement

Outcome	Measure	Data source
Change in protocol compliance (primary outcome)	Percent difference in protocol compliance with mNavigator and historical compliance. Protocol compliance is calculated as proportion of protocol steps completed, based on a compliance checklist.	mNavigator data from prospectively registered pediatric cancer patients from July 2019 – July 2020 and data extracted from paper charts for patients registered at BMC from 2015 – June 2019 entered into mNavigator
Change in treatment abandonment (secondary outcome)	Calculated as the difference in proportion of patients registered in mNavigator who abandoned treatment compared to historical controls who abandoned treatment. Treatment abandonment is defined as missing four or more consecutive weeks of treatment or follow-up while still on therapy.	Data from mNavigator (outcome form)
Change in treatment completion rate	Calculated as the proportion of patients registered in mNavigator who completed treatment compared to historical controls	Data from mNavigator (outcome form)
Change in time to diagnosis	Change in the number of days to diagnosis using mNavigator compared to historical controls. Time to diagnosis is computed as the duration from registration to diagnosis	Data from mNavigator (registration form; diagnosis form)

**Table 4:** System-level outcomes related to the mNavigator application

Outcome	Measure	Data source
Usability^a^	System usability scale score ranging from 0 to 100. A SUS score above a 68 is considered above average and anything below 68 is below average.	10-point validated system usability scale
Acceptability	Proportion of providers who would continue to use the app. Proportion of providers who would recommend the app to others.	Semi-structured interviews
Utilization	Number of forms submitted, stratified, by users, per month of implementation	Data from mNavigator (Mobile users statistics)
Reach	Number of patients registered in mNavigator during study period	Data from mNavigator (Mobile users statistics)
Stability	• Number of instances of mNavigator failure per month (all-causes) • Number of instances of CommCare failure per month (all-causes) • Number of instances of device • failure per month (all-causes)	Data from CommCare (Worker Activity and Daily Activity reports) cross checked with manual reports from Mobile users
Training	Number of hours of initial training as well as hours of ongoing support provided during the first month of Implementation	Manually recorded from Duke team members that are performing initial training
User-proficiency	Number of users who are proficient in use of mNavigator within first month of implementation	Data from CommCare (Worker Activity reports)
Time per form	Average time in minutes spent completing each form, stratified by form	Data from mNavigator (Mobile users statistics)
Time per patient	Total time in minutes spent entering patient data in mNavigator, from time of registration until an outcome is recorded. Calculated by summing time for completing each form by patient.	Data from mNavigator (Mobile users statistics)

^a^System Usability Scale developed by John Brooke is available from www.usability.gov

### Statistical analysis

#### Quantitative analysis plan

Descriptive statistical measures (e.g., frequencies, means, proportions, etc.) will be generated using STATA (v15 or higher) to describe basic socio-demographic and clinical profiles of study participants. A compliance score will be generated based on the proportion of protocol steps completed. Difference-in-difference (DID) estimation will be used to track longitudinal differences in compliance from baseline to end line at BMC. For secondary outcomes, logistic regression will be used to assess provider characteristics associated with protocol compliance and completion of critical steps in the checklist. Patient characteristics at BMC will be compared using χ2 tests (binary variable) and t-tests (continuous variables).

#### Qualitative analysis plan

For observations and in-depth interviews conducted with health providers and staff, we will use applied thematic analysis on the observation notes and interview transcripts. Electronic files may be uploaded into QSR NVivo software (v12 or higher) that supports coding and finer level re-coding of text data that enables researchers to explore how concepts fit by developing and modifying a hierarchical coding index. Thematic analysis will be conducted via an iterative process of data collection and analysis that utilizes four interrelated steps: reading, coding, data display, and data reduction. The team will use a codebook of a priori, structural codes consistent with CFIR and based on the observation and interview guides. A second round of coding, i.e. content coding, will be conducted to identify additional themes, ideas, or concepts. Twenty percent of transcripts will be coded by two team members to assess inter-rater reliability. Discussions will be held to resolve coding discrepancies. We may generate summaries of interviews and look across interviews for commonly named problems and solutions related to mNavigator. Data from the observations will be summarized as workflow diagrams, tables or other visual or narrative summaries to describe domains that help assess reach, effectiveness, adoption, implementation, and technical functionality and stability [28].

## Discussion

To our knowledge, mNavigator is the first digital health case management system specifically developed to improve health provider compliance with pediatric cancer treatment protocols in a low-resource setting.

The study has several strengths. First, mNavigator bolsters current standard of care by facilitating compliance with clinical best practices, promoting care coordination, and allowing simultaneous, ongoing quality of care evaluation. Currently at cancer centers in Tanzania, there is no mechanism to facilitate or monitor implementation of protocol-driven treatment in real time. Static paper-based medical records further prevent proactive actions to reduce treatment abandonment in pediatric cancer patients. A digital case management system such as mNavigator can dynamically identify and highlight instances of poor protocol compliance, errors in diagnosis or treatment, or patients lost to follow-up as compared to paper-based systems, which would require greater investments of human resources and time. By supporting allied health workers (nurses and patient navigator) in documenting patient cancer diagnosis and treatment data in mNavigator, patient-specific treatment plans are generated by the system which allows the implementation of treatment protocols via algorithms, checklists and alerts. As such, mNavigator promotes task shifting of lower-priority clinical tasks (e.g., measuring patients height/weight, data entry etc.) to ensure that the limited supply of highly trained physicians are used as efficiently as possible. This could reduce the clinical workflow burden for trained physicians and increase protocol compliance, reduce diagnostic delays, and improve quality of patient care.

Second, mNavigator seeks to strengthen health system capacity as the foundation for strategies targeting patient-centered barriers to pediatric cancer treatment abandonment. While patient-centered barriers contribute to treatment abandonment, reducing these barriers alone is inadequate if health system inefficiencies prevent the provision of quality and timely services to those who access healthcare. We posit that, by targeting health system inefficiencies first, we can more effectively set the stage for reducing patient-centered barriers, including via use of patient-facing digital health strategies (e.g., health education, engagement in care between treatment visits, appointment reminders etc.). This health system focus is innovative for treatment abandonment since most current efforts focus solely on patient-level barriers without regard to resource-constraints that are common to health systems in LMICs.

Third, our study leverages specific advantages of mobile technologies in LMICs. While tethered systems (e.g., desktops) can support much of the functionality proposed in the study, the higher costs of purchasing and maintaining desktops, and the need for constant power supply make them less feasible in LMICs where financial resources and reliable electricity are often scarce. In contrast, the affordability and exponential growth in ownership and mobile broadband subscriptions, makes mobile devices the information and communication technology of choice in LMICs [30]. There are currently two desktops in the oncology division at BMC which are used for research projects (not patient management), but 100% of the staff have smart phones, meaning a digital case management system can be broadly implemented across different cadres of providers using mobile devices. The ability to collect and manage data offline through digital health platforms optimized for LMICs, and touch screen interface, are other unique advantages over desktops. The portability of mobile devices, which can be temporarily used offline, allows providers to access case data and treatment guidelines throughout the hospital, at home, or at satellite clinics, increasing opportunities for appropriate protocol use. This has implications for future studies targeting outreach activities at BMC where at-risk pediatric cancer patients may be identified for follow up in community-settings.

Finally, it mitigates concerns related to scalability of mHealth interventions by: adapting a validated, open-source mobile platform to reduce risks in the system development lifecycle and improve generalizability; using a theory-driven implementation science approach to improve contextual relevance and acceptability; and, building clinical, research, and digital health capacity at Tanzanian cancer centers, so that this and future digital interventions can be supported locally. BMC would serve as a pilot site with potential for expansion and multisite clinical research infrastructure development across Tanzania. In doing so, this study addresses criticisms of digital health systems related to poor contextual relevance, custom closed-source systems that lack inter-operability, and failure to develop appropriate local human resource capacity needed for system longevity.

Study limitations include the small sample size of mNavigator users as well as the prospective and historical patients proposed to be included in the evaluation. Since study implementation is limited to one site (BMC) based on budget and other resources available, the study sample size is limited by the availability of individuals who can participate in the study. The decision to use historical, instead of contemporary controls is also motivated by the same constraints.

Results from the evaluation of mNavigator will inform implementation and evaluation of the system in other tertiary pediatric cancer facilities in Tanzania, with the goal to facilitate protocol-driven treatment. Study findings will inform core components and elements of mNavigator, which can be adapted for use in different settings.

## Supplementary information

**Additional file 1: Table S1**: SPIRIT 2013 Checklist: Recommended items to address in a clinical trial protocol and related documents*. **Table S2:** WHO Trial Registration Data Set. **Table S3** Available resources. **Table S4** Application of Consolidated Framework for Implementation Research Constructs to the development and implementation of mNavigator.

**Additional file 2.** Example informed consent form for caregivers of children approached for participation in the study.

## Data Availability

The data that support the findings of this study are available from the Bugando Medical Centre (BMC) but restrictions apply to the availability of these data, which were used under license for the current study, and so are not publicly available.

## Abbreviations

BL: Burkitt lymphoma
BMC: Bugando medical centre
CFIR: Consolidated framework for implementation research
DID: Difference-in-difference
HIC: High-income countries
HIPAA: Health insurance portability and accountability act
HQ: Headquarters
IT: Information technology
LMIC: Low- and middle-income countries
ORIC: Organizational readiness for implementing change
Rb: Retinoblastoma
RE-AIM: Reach, effectiveness, adoption, implementation and maintenance framework
TiDieR: Template for intervention description and replication
