# Supplementary material for: Using digital health to facilitate compliance with standardized pediatric cancer treatment guidelines in Tanzania: protocol for an early-stage effectiveness-implementation hybrid study
Source: BMC Cancer. 2020 Mar 29;20:254. doi: 10.1186/s12885-020-6611-3 (PMC7104518; doi:10.1186/s12885-020-6611-3)
Supplement: Supplementary file 1 — Additional file 1: Table S1: SPIRIT 2013 Checklist: Recommended items to address in a clinical trial protocol and related documents*. Table S2: WHO Trial Registration Data Set. Table S3 Available resources. Table S4 Application of Consolidated Framework for Implementation Research Constructs to the development and implementation of mNavigator. [file 12885_2020_6611_MOESM1_ESM.docx]

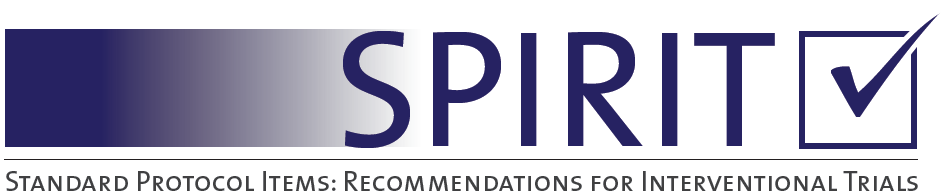


Table S1: SPIRIT 2013 Checklist: Recommended items to address in a clinical trial protocol and related documents*

| Section/item | ItemNo | Description | Location in manuscript |
| --- | --- | --- | --- |
| **Administrative information** | | |  |
| Title | 1 | Descriptive title identifying the study design, population, interventions, and, if applicable, trial acronym | Title page, pg 8 |
| Trial registration | 2a | Trial identifier and registry name. If not yet registered, name of intended registry | Abstract |
|  | 2b | All items from the World Health Organization Trial Registration Data Set | Table S2 |
| Protocol version | 3 | Date and version identifier | Not applicable |
| Funding | 4 | Sources and types of financial, material, and other support | Declarations-funding |
| Roles and responsibilities | 5a | Names, affiliations, and roles of protocol contributors | Title page, declarations – author contributions |
|  | 5b | Name and contact information for the trial sponsor | Table S2 |
|  | 5c | Role of study sponsor and funders, if any, in study design; collection, management, analysis, and interpretation of data; writing of the report; and the decision to submit the report for publication, including whether they will have ultimate authority over any of these activities | Declarations-funding |
|  | 5d | Composition, roles, and responsibilities of the coordinating centre, steering committee, endpoint adjudication committee, data management team, and other individuals or groups overseeing the trial, if applicable (see Item 21a for data monitoring committee) | Not applicable |
| Introduction |  |  |  |
| Background and rationale | 6a | Description of research question and justification for undertaking the trial, including summary of relevant studies (published and unpublished) examining benefits and harms for each intervention | Background, pp 7-8 |
|  | 6b | Explanation for choice of comparators | Pg 17 |
| Objectives | 7 | Specific objectives or hypotheses | Study aims, pp 8-9 |
| Trial design | 8 | Description of trial design including type of trial (eg, parallel group, crossover, factorial, single group), allocation ratio, and framework (eg, superiority, equivalence, noninferiority, exploratory) | Title, Methods pg 16 |
| Methods: Participants, interventions, and outcomes | | |  |
| Study setting | 9 | Description of study settings (e.g., community clinic, academic hospital) and list of countries where data will be collected. Reference to where list of study sites can be obtained | Pg 9 |
| Eligibility criteria | 10 | Inclusion and exclusion criteria for participants. If applicable, eligibility criteria for study centres and individuals who will perform the interventions (eg, surgeons, psychotherapists) | Pp 19-20 |
| Interventions | 11a | Interventions for each group with sufficient detail to allow replication, including how and when they will be administered | Pp 9 - 13 |
|  | 11b | Criteria for discontinuing or modifying allocated interventions for a given trial participant (eg, drug dose change in response to harms, participant request, or improving/worsening disease) | Not applicable |
|  | 11c | Strategies to improve adherence to intervention protocols, and any procedures for monitoring adherence (eg, drug tablet return, laboratory tests) | Not applicable |
|  | 11d | Relevant concomitant care and interventions that are permitted or prohibited during the trial | Not applicable |
| Outcomes | 12 | Primary, secondary, and other outcomes, including the specific measurement variable (eg, systolic blood pressure), analysis metric (eg, change from baseline, final value, time to event), method of aggregation (eg, median, proportion), and time point for each outcome. Explanation of the clinical relevance of chosen efficacy and harm outcomes is strongly recommended | Pg 21, Tables 3 & 4 |
| Participant timeline | 13 | Time schedule of enrolment, interventions (including any run-ins and washouts), assessments, and visits for participants. A schematic diagram is highly recommended (see Figure) | Pg 17 |
| Sample size | 14 | Estimated number of participants needed to achieve study objectives and how it was determined, including clinical and statistical assumptions supporting any sample size calculations | Pg 20-21 |
| Recruitment | 15 | Strategies for achieving adequate participant enrolment to reach target sample size | Pg 20-21 |
| **Methods: Assignment of interventions (for controlled trials)** | | |  |
| Allocation: |  |  |  |
| Sequence generation | 16a | Method of generating the allocation sequence (eg, computer-generated random numbers), and list of any factors for stratification. To reduce predictability of a random sequence, details of any planned restriction (eg, blocking) should be provided in a separate document that is unavailable to those who enrol participants or assign interventions | Not applicable |
| Allocation concealment mechanism | 16b | Mechanism of implementing the allocation sequence (eg, central telephone; sequentially numbered, opaque, sealed envelopes), describing any steps to conceal the sequence until interventions are assigned | Not applicable |
| Implementation | 16c | Who will generate the allocation sequence, who will enrol participants, and who will assign participants to interventions | Not applicable |
| Blinding (masking) | 17a | Who will be blinded after assignment to interventions (eg, trial participants, care providers, outcome assessors, data analysts), and how | Not applicable |
|  | 17b | If blinded, circumstances under which unblinding is permissible, and procedure for revealing a participant’s allocated intervention during the trial | Not applicable |
| **Methods: Data collection, management, and analysis** | | |  |
| Data collection methods | 18a | Plans for assessment and collection of outcome, baseline, and other trial data, including any related processes to promote data quality (eg, duplicate measurements, training of assessors) and a description of study instruments (eg, questionnaires, laboratory tests) along with their reliability and validity, if known. Reference to where data collection forms can be found, if not in the protocol | Data is collected using mNavigator |
|  | 18b | Plans to promote participant retention and complete follow-up, including list of any outcome data to be collected for participants who discontinue or deviate from intervention protocols | Data is collected using mNavigator |
| Data management | 19 | Plans for data entry, coding, security, and storage, including any related processes to promote data quality (eg, double data entry; range checks for data values). Reference to where details of data management procedures can be found, if not in the protocol | Data is collected using mNavigator. Data security and storage details are listed on pg 10. Data QA procedures are described on pp 18-19 |
| Statistical methods | 20a | Statistical methods for analysing primary and secondary outcomes. Reference to where other details of the statistical analysis plan can be found, if not in the protocol | Pp 21-22 |
|  | 20b | Methods for any additional analyses (eg, subgroup and adjusted analyses) | Qualitative data analysis is described on pp 22-23 |
|  | 20c | Definition of analysis population relating to protocol non-adherence (eg, as randomised analysis), and any statistical methods to handle missing data (eg, multiple imputation) | Not applicable |
| **Methods: Monitoring** | | |  |
| Data monitoring | 21a | Composition of data monitoring committee (DMC); summary of its role and reporting structure; statement of whether it is independent from the sponsor and competing interests; and reference to where further details about its charter can be found, if not in the protocol. Alternatively, an explanation of why a DMC is not needed | Not applicable |
|  | 21b | Description of any interim analyses and stopping guidelines, including who will have access to these interim results and make the final decision to terminate the trial | Not applicable |
| Harms | 22 | Plans for collecting, assessing, reporting, and managing solicited and spontaneously reported adverse events and other unintended effects of trial interventions or trial conduct | Not applicable |
| Auditing | 23 | Frequency and procedures for auditing trial conduct, if any, and whether the process will be independent from investigators and the sponsor | Pg 18 |
| Ethics and dissemination | | |  |
| Research ethics approval | 24 | Plans for seeking research ethics committee/institutional review board (REC/IRB) approval | Declarations – ethics approval |
| Protocol amendments | 25 | Plans for communicating important protocol modifications (eg, changes to eligibility criteria, outcomes, analyses) to relevant parties (eg, investigators, REC/IRBs, trial participants, trial registries, journals, regulators) | Declarations – ethics approval |
| Consent or assent | 26a | Who will obtain informed consent or assent from potential trial participants or authorised surrogates, and how (see Item 32) | Pg 16 |
|  | 26b | Additional consent provisions for collection and use of participant data and biological specimens in ancillary studies, if applicable | Not applicable |
| Confidentiality | 27 | How personal information about potential and enrolled participants will be collected, shared, and maintained in order to protect confidentiality before, during, and after the trial | Supplementary file 5 |
| Declaration of interests | 28 | Financial and other competing interests for principal investigators for the overall trial and each study site | Declarations – competing interests |
| Access to data | 29 | Statement of who will have access to the final trial dataset, and disclosure of contractual agreements that limit such access for investigators | Declarations – data availability statement |
| Ancillary and post-trial care | 30 | Provisions, if any, for ancillary and post-trial care, and for compensation to those who suffer harm from trial participation | Not applicable |
| Dissemination policy | 31a | Plans for investigators and sponsor to communicate trial results to participants, healthcare professionals, the public, and other relevant groups (eg, via publication, reporting in results databases, or other data sharing arrangements), including any publication restrictions | Supplementary file 5 |
|  | 31b | Authorship eligibility guidelines and any intended use of professional writers | Not applicable |
|  | 31c | Plans, if any, for granting public access to the full protocol, participant-level dataset, and statistical code | Declarations-data availability statement |
| Appendices |  |  |  |
| Informed consent materials | 32 | Model consent form and other related documentation given to participants and authorised surrogates | Supplementary methods |
| Biological specimens | 33 | Plans for collection, laboratory evaluation, and storage of biological specimens for genetic or molecular analysis in the current trial and for future use in ancillary studies, if applicable | Not applicable |

*It is strongly recommended that this checklist be read in conjunction with the SPIRIT 2013 Explanation & Elaboration for important clarification on the items. Amendments to the protocol should be tracked and dated. The SPIRIT checklist is copyrighted by the SPIRIT Group under the Creative Commons “[Attribution-NonCommercial-NoDerivs 3.0 Unported](http://www.creativecommons.org/licenses/by-nc-nd/3.0/)” license.

**Table S2: WHO Trial Registration Data Set**

| **Data category** | **Information** |
| --- | --- |
| Primary registry and trial identifying number | ClinicalTrials.gov  NCT03677128 |
| Date of registration in primary registry | September 19, 2018 |
| Secondary identifying numbers | R21CA217268-01A1 |
| Source(s) of monetary or material support | National Cancer Institute (NCI) |
| Primary sponsor | Duke University, USA |
| Secondary sponsor(s) | None |
| Contact for public queries | Kristin Schroeder, MD, MPH  Ph: 919-668-6288  [kristin.schroeder@duke.edu](mailto:kristin.schroeder@duke.edu) |
| Contact for scientific queries | Kristin Schroeder, MD, MPH  Ph: 919-668-6288  [kristin.schroeder@duke.edu](mailto:kristin.schroeder@duke.edu)  Lavanya Vasudevan, PhD, MPH  Ph: 9196131423  [lavanya.vasudevan@duke.edu](mailto:lavanya.vasudevan@duke.edu) |
| Public title | Mobile Health Case Management System for Reducing Pediatric Treatment Abandonment |
| Scientific title | Mobile Health Case Management System for Reducing Pediatric Treatment Abandonment |
| Countries of recruitment | Tanzania |
| Health condition(s) or problem(s) studied | Pediatric Cancer (Burkitt Lymphoma, Retinoblastoma) |
| Intervention(s) | Behavioral: mNavigator digital case management system |
| Key inclusion and exclusion criteria (for ca | There are two categories of participants: Patients with Burkitt Lymphoma or retinoblastoma; and health providers at BMC who participate in testing and/or use of mNavigator.  Eligibility criteria are as follows:  A) For patients:  *All patients will be registered in the pre-diagnosis cohort but, for the purposes of this study, primary and secondary outcomes will only be tracked for patients with BL or RB once the diagnosis is made.  Inclusion Criteria:   - Pediatric oncology patients diagnosed with Burkitt Lymphoma or Retinoblastoma under the age of 18   Exclusion criteria:   - Patients older than 18 years at registration - Patients with diagnoses other than Burkitt lymphoma or retinoblastoma.   B) For providers:  Inclusion Criteria:   - Must be health provider or staff working at BMC who provides care for cancer patients. - Must be 18 years of age or older.   Exclusion:   - Persons younger than 18 years of age. |
| Study type | Interventional (Clinical Trial) |
| Date of first enrolment | July 23, 2019 |
| Target sample size | 65 |
| Recruitment status | Open for recruitment |
| Primary outcome(s) | 1. Protocol compliance: Percentage difference in protocol compliance with mNavigator and historical compliance. [Time Frame: Approximately 1 year] |
| Key secondary outcomes | 1. Time to diagnosis (in days): The number of days to diagnosis using mNavigator compared to historical controls. Time to diagnosis is computed as the duration (in days) from registration to diagnosis. [Time Frame: Approximately 1 year] 2. Treatment abandonment: Calculated as the difference in proportion of patients registered in mNavigator who abandon treatment compared to historical controls who abandon treatment. Treatment abandonment is defined as missing 4 or more consecutive weeks of treatment or follow-up while on therapy. [Time Frame: Approximately 1 year] |

**Table S3: Available resources**

| **Resources** | **Existing** | **Study-supported** |
| --- | --- | --- |
| Electricity | X |  |
| Network connectivity | X |  |
| Patient navigators | X | X |
| Server space |  | X |
| Programmer Time |  | X |
| Tablet devices |  | X |
| CommCare subscription |  | X |

| **Table S4: Application of Consolidated Framework for Implementation Research Constructs to the development and implementation of mNavigator.** | | | |
| --- | --- | --- | --- |
| **Construct** | | **Short Description** | **mNavigator** |
| **I. INTERVENTION CHARACTERISTICS** | | | |
| A | Intervention Source | Perception of key stakeholders about whether the intervention is externally or internally developed. | Internally developed by a pediatric oncologist who works at BMC, and with BMC providers and staff as stakeholders. |
| B | Evidence Strength & Quality | Stakeholders’ perceptions of the quality and validity of evidence supporting the belief that the intervention will have desired outcomes. | mNavigator development based on documented challenges in pediatric patient care and evidence for high treatment abandonment rates at BMC. Protocol content is based on clinical guidelines developed / adopted by Tanzania Ministry of Health and nationally-approved for use. |
| C | Relative Advantage | Stakeholders’ perception of the advantage of implementing the intervention versus an alternative solution. | Will be assessed as part of usability testing of mNavigator. Comparison is the use of non-digital client health records. |
| D | Adaptability | The degree to which an intervention can be adapted, tailored, refined, or reinvented to meet local needs. | - Incorporates resource-adapted treatment guidelines that are approved by the Tanzanian Ministry of Health. - CommCare platform highly suitable for resource-constrained setting due to ease of use, offline access, user-interface friendly to low digital literacy populations, etc. - Can accommodate use by a flexible workforce including patient navigators by incorporating their workflow in the design of mNavigator. |
| E | Trialability | The ability to test the intervention on a small scale in the organization, and to be able to reverse course (undo implementation) if warranted. | - Evaluating usability with end-users at BMC. - Evaluating efficacy of mNavigator in small patient sample (n=50). |
| F | Complexity | Perceived difficulty of implementation, reflected by duration, scope, radicalness, disruptiveness, centrality, and intricacy and number of steps required to implement. | Will be determined as part of usability and acceptability assessments. Initial design has been focused on reducing complexity of treatment protocols by the use of decision support, checklists, automated calculations, skip logic, and prompts. |
| H | Cost | Costs of the intervention and costs associated with implementing the intervention including investment, supply, and opportunity costs. | Time (providers’ time using mNavigator, programming time), software, and programming costs measured will be reported. |
| **II. OUTER SETTING** | |  |  |
| A | Patient Needs & Resources | The extent to which patient needs, as well as barriers and facilitators to meet those needs, are accurately known and prioritized by the organization. | Addresses diagnostic delays, shown to effect patient outcomes at BMC in prior research. Additionally includes cancer and treatment education videos for families to improve knowledge about cancer, a key barrier to completing cancer treatment. |
| B | Cosmopolitanism | The degree to which an organization is networked with other external organizations. | BMC one of three referral hospitals for pediatric cancer in Tanzania. Providers from the three organizations are part of the T-POT consortium. |
| C | Peer Pressure | Mimetic or competitive pressure to implement an intervention; typically because most or other key peer or competing organizations have already implemented or are in a bid for a competitive edge. | Not relevant. No competing programs in Tanzania. |
| D | External Policy & Incentives | A broad construct that includes external strategies to spread interventions, including policy and regulations (governmental or other central entity), external mandates, recommendations and guidelines, pay-for-performance, collaboratives, and public or benchmark reporting. | Protocol content is based on clinical guidelines developed / adopted by Tanzania Ministry of Health and nationally-approved for use. |
| **III. INNER SETTING** | |  |  |
| A | Structural Characteristics | The social architecture, age, maturity, and size of an organization. | BMC oncology department started in 2009, with development of pediatric research collaboration in 2014. It is one of three hospitals in the country that treats pediatric cancer. |
| B | Networks & Communications | The nature and quality of webs of social networks and the nature and quality of formal and informal communications within an organization. | High speed internet is available. Formal communication is done through weekly tumor board, and digital group messaging (WhatsApp, phone texts) is routinely used for informal patient care coordination. |
| C | Culture | Norms, values, and basic assumptions of a given organization. | BMC as a tertiary referral hospital exists to improve the welfare of the community in the Lake Zone through provision of state of the art, diversified, specialized and super specialized health care, research, training and consultancy services guided by Catholic Church ethics and morals. |
| D | Implementation Climate | The absorptive capacity for change, shared receptivity of involved individuals to an intervention, and the extent to which use of that intervention will be rewarded, supported, and expected within their organization. | Measured in a previous study. High mobile phone ownership and literacy. |
| 1 | Tension for Change | The degree to which stakeholders perceive the current situation as intolerable or needing change. | Measured in a previous study. High organizational readiness to change. |
| 2 | Compatibility | The degree of tangible fit between meaning and values attached to the intervention by involved individuals, how those align with individuals’ own norms, values, and perceived risks and needs, and how the intervention fits with existing workflows and systems. | System based on extensive workflow mapping to describe existing processes. ‘Fit’ with clinical workflows also assessed during usability testing. |
| 3 | Relative Priority | Individuals’ shared perception of the importance of the implementation within the organization. | Shared commitment to reduce tx abandonment and this is perceived as an important priority to achieve that goal. |
| E | Readiness for Implementation | Tangible and immediate indicators of organizational commitment to its decision to implement an intervention. | Measured in a previous study. High organizational readiness to change. |
| 1 | Leadership Engagement | Commitment, involvement, and accountability of leaders and managers with the implementation. | Co-investigators from BMC participating in study. Letter of support indicating commitment to the project from BMC leadership. |
| 2 | Available Resources | The level of resources dedicated for implementation and on-going operations, including money, training, education, physical space, and time. | See Table S1 Existing resources are expected to continue to be available after the study ends. |
| 3 | Access to Knowledge & Information | Ease of access to digestible information and knowledge about the intervention and how to incorporate it into work tasks. | Available during training of end-users prior to implementation, and as in-app prompts. |
| **IV. CHARACTERISTICS OF INDIVIDUALS** | | |  |
| B | Self-efficacy | Individual belief in their own capabilities to execute courses of action to achieve implementation goals. | Measured in a previous study. High mobile phone ownership and literacy among providers. |
| C | Individual Stage of Change | Characterization of the phase an individual is in, as he or she progresses toward skilled, enthusiastic, and sustained use of the intervention. | Measured in a previous study, 100% of providers thought that it would be useful to use a mobile phone to communicate with patients, share lab results, and view standardized pediatric treatment protocols/checklists. |
| **V. PROCESS** | |  |  |
| A | Planning | The degree to which a scheme or method of behavior and tasks for implementing an intervention are developed in advance, and the quality of those schemes or methods. | Assessed in this protocol paper |
| B | Engaging | Attracting and involving appropriate individuals in the implementation and use of the intervention through a combined strategy of social marketing, education, role modeling, training, and other similar activities. | Assessed during pre-implementation training |
| 1 | Opinion Leaders | Individuals in an organization who have formal or informal influence on the attitudes and beliefs of their colleagues with respect to implementing the intervention. | Assessed during pre-implementation training |
| 2 | Formally Appointed Internal Implementation Leaders | Individuals from within the organization who have been formally appointed with responsibility for implementing an intervention as coordinator, project manager, team leader, or other similar role. | Principal Investigator at BMC |
| 3 | Champions | “Individuals who dedicate themselves to supporting, marketing, and ‘driving through’ an [implementation]” [101] (p. 182), overcoming indifference or resistance that the intervention may provoke in an organization. | Study principal investigator who also works at BMC as a pediatric oncologist. |
| 4 | External Change Agents | Individuals who are affiliated with an outside entity who formally influence or facilitate intervention decisions in a desirable direction. | Stakeholders at the Muhimbili National Hospital. |
| C | Executing | Carrying out or accomplishing the implementation according to plan. | Assessed as intervention fidelity and compliance with standardized treatment guidelines. |
| D | Reflecting & Evaluating | Quantitative and qualitative feedback about the progress and quality of implementation accompanied with regular personal and team debriefing about progress and experience. | Achieved through regular team meetings, data collection on system functionality, and intervention fidelity. |
|  |  |  |  |

**Note:** The following constructs are not assessed in the study. **I. Intervention characteristics:** Design quality and packaging. **III. Inner setting:** Organizational incentives and rewards, goals and feedback, learning climate. **IV. Characteristics of individuals:** A. Knowledge and beliefs about the intervention, individual identification with organization, other personal attributes.
