# Supplementary material for: Using digital health to facilitate compliance with standardized pediatric cancer treatment guidelines in Tanzania: protocol for an early-stage effectiveness-implementation hybrid study
Source: BMC Cancer. 2020 Mar 29;20:254. doi: 10.1186/s12885-020-6611-3 (PMC7104518; doi:10.1186/s12885-020-6611-3)
Supplement: Supplementary file 2 — Additional file 2. Example informed consent form for caregivers of children approached for participation in the study. [file 12885_2020_6611_MOESM2_ESM.docx]

**Additional file 2: Example informed consent form for caregivers of children approached for participation in the study.**

Form M0345

**BUGANDO MEDICAL CENTRE**


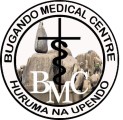

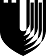
**DUKE UNIVERSITY HEALTH SYSTEM**

**Study ID:** | | |B|M|C| | | |

**Study Title:** A Digital Case Management System for Reducing Pediatric Cancer Treatment Abandonment in Tanzania

**Consent to Participate in a Research Study**

**CONCISE SUMMARY**

We are a group of researchers from Bugando Medical Centre (Tanzania) and Duke University (United States) who are studying children’s cancers, In particular, we are testing a tool, called mNavigator, which will guide health providers follow steps of cancer treatment as described in Tanzanian national guidelines. The study is also looking to better understand how we can help patients start and complete their cancer treatment. Dr. Kristin Schroeder and Dr. Lavanya Vasudevan from Duke University and Dr. Nestory Masalu from Bugando Medical Centre are leading this study.

The main goals of the study are:

1. To develop a digital case management program on Tanzanian childhood cancer treatment protocols.
2. To evaluate if this program can improve provider compliance with standardized childhood cancer treatment protocols and reducing treatment abandonment.

A secondary goal of the study is to understand what the barriers are and what will make it easier for other referral hospitals to use the mNavigator program to treat children with cancer.

You have been participating in this study with the consent provided by your parent or caregiver, and your assent. Now that you have turned 18 years of age, we are asking you to confirm that you are still interested in continuing your participation in this study.

Because you have been diagnosed with Burkitt lymphoma or retinoblastoma, you are eligible to participate in this study. Specifically, we are asking that:

- - You participate by letting us record all of your medical information related to your cancer care into mNavigator so that we can follow how health professionals use the system to guide cancer treatment. Irrespective of whether or not you choose to participate in this study, your treatment will not be affected. You will receive all treatment that you are entitled to.

It is important for you to know that participation in this study is voluntary which means that:

- - You can choose whether or not you would like to be in this study.
  - You can decide to stop participating in the study at any time.
  - Your decision to participate in this study or not will have no effect on your medical care.

There are no significant risks to participating in this study. A potential benefit of participation is that providers using mNavigator may follow national guidelines for treatment better, and so you may receive treatment that is more consistent with national guidelines and help us provide you better quality care. Although this cannot be guaranteed, following national treatment guidelines are expected to improve the results of treatment.

*DUHS IRB*

*IRB NUMBER: Pro00094010*

*IRB REFERENCE DATE: 10/08/2019 IRB EXPIRATION DATE: 04/30/2021*

Page 1 of 6 Subject Initials: Consent_33334.doc

Form M0345

**BUGANDO MEDICAL CENTRE**


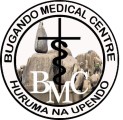

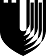
**DUKE UNIVERSITY HEALTH SYSTEM**

**Study ID:** | | |B|M|C| | | |

**Study Title:** A Digital Case Management System for Reducing Pediatric Cancer Treatment Abandonment in Tanzania

Approximately 50 patients and their parents or guardians will participate in this study. Participation will last the duration of cancer treatment and follow-up.

The information just described will be discussed in greater detail in this consent form. Study staff will give you a copy of the consent form with a contact phone number to call in case you have questions or concerns about the study. As you decide whether or not to participate in this study, please ask the study staff any questions you have about the study.

**INTRODUCTION**

Researchers at Duke University have partnered with Bugando Medical Centre (BMC) in Mwanza to conduct research on children’s cancers, with a focus on increasing treatment compliance and reducing treatment abandonment. As part of the study, we are developing a digital case management system, called mNavigator, which uses two nationally-approved treatment protocols, one for Burkitt lymphoma (BL) and the other for retinoblastoma (Rb). We will test mNavigator which will be used to facilitate protocol-driven treatment and improve health provider compliance with these standardized treatment protocols. Dr. Kristin Schroeder and Dr. Lavanya Vasudevan from Duke University and Dr. Nestory Masalu from Bugando Medical Centre will conduct the study. A grant from the National Cancer Institute, USA is funding this study. Portions of the study leaders’ (Drs. Schroeder, Masalu and Vasudevan) and study staff salaries will be paid by the grant.

Research studies are voluntary and include only people who choose to take part. Please read this consent form carefully or have study staff read to you, and take your time making your decision. As study staff discuss this consent form with you, please ask them to explain any words or information that you do not clearly understand. We encourage you to talk with your family and friends before you decide to take part in this research study. The nature of the study, risks, inconveniences, discomforts, and other important information are listed below.

**WHY IS THIS STUDY BEING DONE?**

This study is being done because in the past only 50% of patients completed treatment. In other studies, it has been shown that when health providers use standardized treatment protocols, more children complete treatment. Although Tanzania has national guidelines and protocols related to cancer treatment, health providers at BMC do not have tools that can help them follow the national guidelines and protocols. This study develops and tests mNavigator as a tool to help health providers follow steps of cancer treatment as described in Tanzanian national guidelines.

**WHO WILL BE MY DOCTORS ON THIS STUDY?**

If you decide to participate, Dr. Kristin Schroeder and Dr. Nestory Masalu will be your doctors for the study and will be in contact with your regular health care provider throughout the time that you are in the study and afterwards, if needed.

*DUHS IRB*

*IRB NUMBER: Pro00094010*

*IRB REFERENCE DATE: 10/08/2019 IRB EXPIRATION DATE: 04/30/2021*

Page 2 of 6 Subject Initials: Consent_33334.doc

Form M0345

**BUGANDO MEDICAL CENTRE**


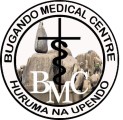

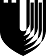
**DUKE UNIVERSITY HEALTH SYSTEM**

**Study ID:** | | |B|M|C| | | |

**Study Title:** A Digital Case Management System for Reducing Pediatric Cancer Treatment Abandonment in Tanzania

**HOW MANY PEOPLE WILL TAKE PART IN THIS STUDY?**

Approximately 50 pediatric cancer patients with diagnosis of either Burkitt lymphoma (BL) or retinoblastoma (Rb) and their parents or guardians will take part in this study at Bugando Medical Centre.

**WHAT IS INVOLVED IN THE STUDY?**

If you agree for you to be in this study, you will be asked to sign this consent form. You are eligible to participate in this study because:

- - You have a known pediatric cancer diagnosis of either Burkitt lymphoma (BL) or retinoblastoma (Rb)
  - You were younger than 18 years of age at enrollment and have now turned 18

Several tests are done routinely as part of your planned standard treatment, and may include laboratory tests such as blood counts and tests to measure the function of the liver and kidneys, a picture of your heart, and x-rays or abdominal ultrasound. As part of the study, you are giving permission to Dr. Schroeder and her study team to record the results of your laboratory tests, imaging, tissue testing and all other information about treatment. This information will be stored in mNavigator.

**HOW LONG WILL I BE IN THIS STUDY?**

We will follow up with you during your routine clinic visits, then again after therapy, for a period of up to 5 years. No additional visits to routine clinic visits are needed for this study. Now that you turned 18 years of age and the study is ongoing, we are re-consenting you as an adult.

**WHAT ARE THE RISKS OF THE STUDY?**

There are no physical risks associated with this study. There is, however, the potential risk of loss of confidentiality. Every effort will be made to keep your information confidential; however, this cannot be guaranteed. Some of the questions we will ask you as part of this study may make you feel uncomfortable. You may refuse to answer any of the questions and you may take a break at any time during the study. You may stop participation in this study at any time. There are no additional risks as a result of your participation in this study.

**ARE THERE BENEFITS TO TAKING PART IN THE STUDY?**

A potential benefit of participation is that providers using mNavigator may follow national guidelines for treatment better, and so you may receive treatment that is more consistent with national guidelines. Although this cannot be guaranteed, following national treatment guidelines are expected to improve the results of treatment. Additionally, we hope that in the future the information learned from this study will benefit other people with your condition.

*DUHS IRB*

*IRB NUMBER: Pro00094010*

*IRB REFERENCE DATE: 10/08/2019 IRB EXPIRATION DATE: 04/30/2021*

Page 3 of 6 Subject Initials: Consent_33334.doc

Form M0345

**BUGANDO MEDICAL CENTRE**


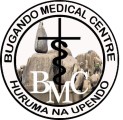

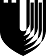
**DUKE UNIVERSITY HEALTH SYSTEM**

**Study ID:** | | |B|M|C| | | |

**Study Title:** A Digital Case Management System for Reducing Pediatric Cancer Treatment Abandonment in Tanzania

**WILL MY INFORMATION BE KEPT CONFIDENTIAL?**

We will do our best to make sure that information about you is kept confidential, but we cannot guarantee total confidentiality. To protect your identity, all identifying information (such as your names, date of birth, or contact information) will be accessed only by authorized study staff at BMC and collaborators at Duke University. In some cases, study records may be reviewed in order to meet federal or state regulations. Reviewers may include representatives and affiliates of Bugando Medical Centre and the Duke University Health System Institutional Review Board, the National Institutes of Health, and others, as appropriate.

Except when required by law or for your care, you will not be identified by name, address, telephone number, or any other direct personal identifier in study records disclosed outside of Bugando Medical Centre.

Since this study is conducted with Duke University, all data from the study will be shared with them. All identifying information collected from you will remain confidential and only BMC or Duke study staff will be able to access it. All data collected on paper will be stored in locked cabinets in secure offices and all electronic data containing identifiable information will be stored on password- protected devices and/or secure devices as approved by regulatory agencies in Tanzania and the United States.

The study results will be retained in your research record for at least six years after the study is completed, or until you turn 21, whichever is longer. At that time, either research information not already in the medical record may be destroyed or information identifying you will be removed from study results at BMC. Any research information in your medical record will be kept indefinitely.

Results of the study will be presented at scientific meetings and published in reports in print or online format. Text, and any pictures or videos published in these reports may be freely available on the internet and may be seen by the general public, including you. Text may also appear on other websites or in print, and may be translated into other languages or used for commercial purposes.

When information from this study is presented at scientific meetings or journals, or elsewhere, you will not be personally identified. By agreeing to be in the study, you consent to publication and presentation of study results.

A description of this study will be available on https:[//www](http://www.clinicaltrials.gov/).c[linicaltrials.gov](http://www.clinicaltrials.gov/) as required by U.S. law. This website will not include information that can identify you. At most, the website will include a summary of the results. You can search this website at any time.

**WHAT ARE THE COSTS?**

There will be no additional costs to you as a result of being in this study. Routine medical care for your condition, that is care you would have received whether or not you were in this study, is not reimbursed by the study team.

*DUHS IRB*

*IRB NUMBER: Pro00094010*

*IRB REFERENCE DATE: 10/08/2019 IRB EXPIRATION DATE: 04/30/2021*

Page 4 of 6 Subject Initials: Consent_33334.doc

Form M0345

**BUGANDO MEDICAL CENTRE**


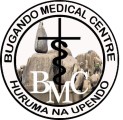

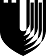
**DUKE UNIVERSITY HEALTH SYSTEM**

**Study ID:** | | |B|M|C| | | |

**Study Title:** A Digital Case Management System for Reducing Pediatric Cancer Treatment Abandonment in Tanzania

**WHAT ABOUT COMPENSATION?**

You will not receive direct compensation for participating in this study.

**WHAT ABOUT RESEARCH RELATED INJURIES?**

Immediate necessary medical care is available at Bugando Medical Centre in the event that you are injured as a result of your participation in this research study. However, there is no commitment by Bugando Medical Centre or its physicians to provide monetary compensation or free medical care to you in the event of a study-related injury.

For questions about the study or research-related injury, contact Dr. Kristin Schroeder at 0686386349.

**WHAT ABOUT MY RIGHTS TO DECLINE PARTICIPATION OR WITHDRAW FROM THE STUDY?**

You may choose not to be in the study. If you agree for you to be in the study, you may withdraw from the study at any time. If you withdraws from the study, no new data about you will be collected for study purposes other than data needed to document your withdrawal. However, any information that you provided to us prior to stopping your participation will be used even if you choose to stop your participation.

Your decision not to participate or to withdraw from the study will not involve any penalty or loss of benefits to which you are entitled, and will not affect your access to health care. If you do not sign this consent form, you will continue to receive care, but not as a part of this study. If you do decide to withdraw, we ask that you inform the study doctors.

We will tell you about new information that may affect your health, welfare, or willingness to stay in this study.

**WHOM DO I CALL IF I HAVE QUESTIONS OR PROBLEMS?**

For questions about the study or a research-related injury, or if you have problems, concerns, questions or suggestions about the research, contact either Dr. Schroeder at 0686386349 or Dr. Masalu at 0783000004 during regular business hours as well as after hours, and on weekends and holidays.

For questions about your rights as a research participant, or to discuss problems, concerns or suggestions related to the research, or to obtain information or offer input about the research, may be addressed to the Chairperson of NatHREC at Tel: +255-22-2121400.

*DUHS IRB*

*IRB NUMBER: Pro00094010*

*IRB REFERENCE DATE: 10/08/2019 IRB EXPIRATION DATE: 04/30/2021*

Page 5 of 6 Subject Initials: Consent_33334.doc

Form M0345

**BUGANDO MEDICAL CENTRE**


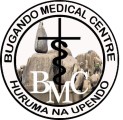

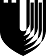
**DUKE UNIVERSITY HEALTH SYSTEM**

**Study ID:** | | |B|M|C| | | |

**Study Title:** A Digital Case Management System for Reducing Pediatric Cancer Treatment Abandonment in Tanzania

**STATEMENT OF CONSENT**

"The purpose of this study, procedures to be followed, risks and benefits have been explained to me. I have been allowed to ask questions, and my questions have been answered to my satisfaction. I have been told whom to contact if I have questions, to discuss problems, concerns, or suggestions related to the research, or to obtain information or offer input about the research. I have read this consent form and agree to be in this study, with the understanding that I may withdraw at any time.

I have been told that I will be given a signed and dated copy of this consent form.

I understand that study findings will be published without my name attached, but that full anonymity cannot be guaranteed. Text, and any pictures or videos published in these reports may be freely available on the internet and may be seen by the general public, including you. The pictures, videos and text may also appear on other websites or in print, and may be translated into other languages or used for commercial purposes. Signing this consent form does not remove my rights to privacy."

**Printed Name of Participant**

**Signature or Fingerprint of Participant Date** (Day/Month/Year) **Time**

*(Witness required if the research participant does not know how to read the consent)*

**Printed Name of Witness**

**Signature of Witness Date** (Day/Month/Year) **Time**

**Signature of Person Obtaining Consent Date** (Day/Month/Year) **Time**

*DUHS IRB*

*IRB NUMBER: Pro00094010*

*IRB REFERENCE DATE: 10/08/2019 IRB EXPIRATION DATE: 04/30/2021*

Page 6 of 6
